# Achieving a ‘Good AI Society’: Comparing the Aims and Progress of the EU and the US

**DOI:** 10.1007/s11948-021-00340-7

**Published:** 2021-11-12

**Authors:** Huw Roberts, Josh Cowls, Emmie Hine, Francesca Mazzi, Andreas Tsamados, Mariarosaria Taddeo, Luciano Floridi

**Affiliations:** 1 Oxford Internet Institute, University of Oxford, 1 St Giles’, Oxford, OX1 3JS UK; 2 Alan Turing Institute, British Library, 96 Euston Rd, London, NW1 2DB UK; 3 Saïd Business School, University of Oxford, Park End St, Oxford, OX1 1HP UK

**Keywords:** Artificial intelligence, European Union, Policy, United States, Social good

## Abstract

Over the past few years, there has been a proliferation of artificial intelligence (AI) strategies, released by governments around the world, that seek to maximise the benefits of AI and minimise potential harms. This article provides a comparative analysis of the European Union (EU) and the United States’ (US) AI strategies and considers (i) the visions of a ‘Good AI Society’ that are forwarded in key policy documents and their opportunity costs, (ii) the extent to which the implementation of each vision is living up to stated aims and (iii) the consequences that these differing visions of a ‘Good AI Society’ have for transatlantic cooperation. The article concludes by comparing the ethical desirability of each vision and identifies areas where the EU, and especially the US, need to improve in order to achieve ethical outcomes and deepen cooperation.

## Introduction

Artificial Intelligence (AI) is a cluster of smart technologies, ranging from machine learning software, to natural language processing applications, to robotics, that has unprecedented capacity to reshape individual lives, societies and the environment (Floridi et al., 2018). Recent years have seen an increasing number of benefits brought about by AI, such as the scientific breakthrough in protein structures made by Deep Mind in 2020 (Callaway, 2020). However, AI applications also come with significant risks. Governmental efforts to govern AI so as to maximise these benefits and minimise risks have only gained substantial traction in the past few years. In a previous article, Cath et al. (2018) undertook an analysis of the AI strategies of the United States (US), the European Union (EU) and the United Kingdom (UK). The article concluded that the documents analysed addressed a variety of ethical, social and economic factors associated with AI, but that none provided an overarching, long-term political vision for the development of a ‘Good AI Society’, nor a clear indication of what such a society would look like in reality.

Since the publication of that article, several high-profile AI documents have been released by all three of the state actors that were considered. Many other states have also demonstrated a keen interest in AI governance, with over 60 releasing AI policy documents (OECD.AI, 2021a, 2021b), a reflection and consequence of the current ‘summer’ which AI is experiencing (Floridi, 2020a; Tsamados et al., 2021). Given these policy developments and the significant societal impact that AI technologies are increasingly having, it is important to revisit these AI strategies to assess their differences and the extent to which long-term visions have been developed. This is the task of the following pages.

Because of the growing global interest in AI governance, which we understand here as the many ways that institutions both private and public, as well as individuals, exercise authority over AI (Yu, 2018), one possible approach would be to expand the analysis beyond the US, EU and UK. Doing so would facilitate a more varied approach on account of the greater breadth of cultural values that are informing other AI strategies (Duan, 2020; Sambasivan et al., 2020). Indeed, we have analysed China’s AI strategy in other articles (Roberts et al., 2021b, 2021c) and many analyses have considered other national approaches elsewhere (Chatterjee, 2020; Cisse, 2018; Gal, 2020). However, in this article, we will focus on two case studies only: the EU and the US. The decision to limit our analysis to two case studies is based on an inevitable trade-off between breadth and depth. We chose to focus on the EU and US in particular because of their global influence over AI governance, which far exceeds other countries (excluding China). More substantively, the EU and the US make for an interesting comparative case study because of their often-touted political alignment over guiding values, such as representative democracy, the rule of law and freedom. Indeed, this alignment has led to widespread calls for deeper transatlantic cooperation in AI governance, particularly in light of the perceived threat that China poses to these values (Delcker, 2020; Lawrence & Cordey, 2020; Meltzer et al., 2020).

Focusing on the AI strategies of the EU and the US leads us to consider two specific research questions. First, which vision of the ‘Good AI Society’ forwarded is more ethically desirable. We consider ethical desirability in terms of the aims outlined and whether these normative visions are being or are likely to be met in practice. Second, given these differing approaches to AI governance, we consider to what extent deep transatlantic cooperation is viable.

Before turning to our analysis, two clarificatory points are required based on this framing. First, policy analyses have already been produced that consider the AI strategies of the EU and of the US in isolation (Brattberg et al., 2020; Rasser, 2019) and comparatively (Allison & Schmidt, 2020; Gill, 2020; Imbrie et al., 2020). However, these analyses have typically^1^ focused only on the capacities of states, including investments in AI, access to data and hardware, and domestic talent, without engaging with these governments’ visions for the role of AI in society, or what we describe here as developing a ‘Good AI Society’. Addressing and contextualising the longer-term, overarching visions that the EU and US have for AI is important, as it provides room for making informed normative judgements about the direction and goals of national strategies.

Second, it is important to expand on what we mean by a ‘Good AI Society’. What constitutes ‘good’ in terms of the development and use of AI is culturally and politically dependent (Floridi et al., 2018). Failing to acknowledge this constitutes a form of absolutism, according to which there is only one, absolutely (i.e., unrelated to any historical or cultural circumstances) ‘valid’ vision, complete and correct, for what would make the use of AI socially ‘good’ (Wong, 2020). At the same time, to consider no values as inherently ‘good’ is a form of extreme metaethical relativism (Brandt, 2001), according to which nothing of substance can ever be said justifiably about the respective merits of different visions. We place ourselves between these two extremes, in the middle ground of ethical pluralism.^2^ This means that we consider there to be many different valid visions of a ‘Good AI Society’, but that each one needs to be underpinned by a set of values that are viewed at national and international levels as desirable. Such values are likely to include democracy,^3^ justice,^4^ privacy,^5^ the protection of human rights,^6^ and a commitment to environmental protection.^7^ Ethical pluralism allows for a limited affirmation of these fundamental values and also an acceptance that values which may (at some higher Level of Abstraction) seem similar can be interpreted and implemented differently across different societies and cultures (Ess, 2020a; Wong, 2020). With an analogy, there are currently 15 types of domestic electrical outlet plugs in use worldwide, they all differ but they are also united by some essential functions and features that make possible adapters and convertors for appliances. Likewise, while some fundamental values may be interpreted similarly in the EU and US, other values may differ, at least in terms of their order of priority and level of importance, and may therefore underlie different visions of a ‘Good AI Society’.

Endorsing a perspective of ethical pluralism allows us to respect these differences and avoid the absolutist trap, but it does not provide guidance on making normative judgements, as there are multiple valid interpretations of fundamental values. Accordingly, another normative step is needed to ensure that meaningful judgements can be made about the different visions for a ‘Good AI Society’ outlined by the EU and US. Our approach is to understand and engage with these visions in a way that accepts a large degree of difference, whilst also using moral reasoning based on our own ethical perspectives to make normative judgements (Angle, 2002). This approach respects difference, but also allows us to undertake an informed analysis of where we perceive interpretations of fundamental values to be ethically undesirable.

Having clarified how we use the expression ‘Good AI Society’, and in light of the gap identified in the literature, in the following pages we compare the development of the governance strategies of these two governments, structuring our analysis around three points:

(i) How the EU and the US conceptualise a ‘Good AI Society’ and the opportunity costs associated with each approach.
(ii) The extent to which the implementation of each vision is living up to stated aims.
(iii) The consequences that these differing visions of a ‘Good AI Society’ have for transatlantic cooperation.

In Sects. “The EU’s Approach to AI” and “The US’s Approach to AI”, we discuss points (i–ii) in regard to the EU’s and US’s AI strategies respectively. We outline how AI policies have evolved and contain distinctive domestic- and international-facing elements. We identify and assess key themes in each, which includes improving economic, social and ethical outcomes domestically; developing a position on AI for defence and international relations externally; and the internal fragmentation of policy outcomes when these strategies are applied in practice. This analysis provides the foundation for answering our first research question of which vision is, in our view, more ethically desirable. In Sect. “Transatlantic Cooperation on AI Governance”, we address point (iii) and consider existing initiatives that promote transatlantic cooperation, as well as barriers to further cooperation that emerge from these differing visions. We conclude by comparing the ethical desirability of the visions and provide a look ahead to the potential for deep transatlantic cooperation around AI governance.

## The EU’s Approach to AI

In May 2016, the EU released its first document addressing the issue of AI governance: a draft report, by the European Parliament’s Committee on Legal Affairs, entitled ‘Civil Law Rules on Robotics’, which called for a coordinated European approach that would employ a mix of hard and soft laws to guard against possible risks. While this report began to address many of the ethical and social issues associated with the development and use of AI, Cath et al. (2018) highlighted a number of gaps, including viewing AI merely as an underlying component of robotics and the failure to acknowledge *accountability* or *transparency* as guiding ethical values.

Since the 2016 JURI report, the focus on AI by EU policymakers has increased significantly. In April 2018, 25 European countries signed a Declaration of Cooperation on Artificial Intelligence,^8^ where they stated their intention to promote a collective European response to AI.^9^ Shortly after, the European Commission published a Communication on the European Approach to AI (‘the Communication’), which outlines a coordinated path forward, centring on the following three priorities:

1. Boosting the EU’s technological and industrial capacity across the economy, in both the private and public sectors.
2. Preparing for the changes brought about by AI through anticipating market change, modernising education and training and adapting social protection systems.
3. Ensuring that there is an appropriate legal and ethical framework that is in line with the EU’s values.

In June 2018, the EU Commission appointed the High-Level Expert Group on AI (HLEG),^10^ a group of 52 experts to support the implementation of the Communication. Amongst other outputs, they produced Ethics Guidelines for AI which focus on developing trustworthy AI by ensuring that systems are lawful, ethical and robust (European Commission, 2019). To achieve aim, seven key requirements are outlined (Table 1), with an accompanying assessment list that offers guidance for practical implementation.

**Table 1:** Requirements for trustworthy AI (European Commission, 2019)

Human agency and oversight	AI systems should allow humans to make informed decisions and be subject to proper oversight
Technical robustness and safety	AI systems need to be resilient, secure, safe, accurate, reliable and reproducible
Privacy and data governance	Adequate data governance mechanisms that fully respect privacy must be ensured
Transparency	The data, system and AI business models should be transparent and explainable to stakeholders
Diversity, non-discrimination and fairness	Unfair bias must be avoided to mitigate the marginalisation of vulnerable groups and the exacerbation of discrimination
Societal and environmental well-being	AI systems should be sustainable and benefit all human beings, including future generations
Accountability	Responsibility and accountability for AI systems and their outcomes should be ensured

Most recently, the European Commission released the draft Artificial Intelligence Act (2021), which proposes a risk-based approach to regulating AI and outlines four categories of risk: unacceptable, high-risk, limited risk and minimal/no risk. Systems deemed to be of unacceptable risk will be prohibited, including cases of social scoring and subliminally manipulative systems (Title II). High-risk AI includes systems which are safety-critical components and those that pose specific risks to fundamental rights. For these systems, specific obligations for providers, importers, distributers, users and authorised representatives are outlined (Title III). Limited risk systems are those that interact with humans, are used for biometric categorisation or generate manipulative content (e.g. deepfakes); these systems have specific transparency requirements (Title IV). For systems which are not high risk, voluntary codes of conduct are encouraged (Title IX). Violating this regulation would lead to fines of up to 6% of global turnover or 30 million euros (Title X).

### Domestic Policy

EU AI policy that has emerged since 2018 addresses many of the key drawbacks highlighted in Cath et al. (2018), including a shift in how AI is conceptualised so as to recognise it as an unembodied technology which ought to be analysed independently from robotics. However, the EU’s definition of AI, as outlined in the proposed AI Act, is extremely broad and includes ‘statistical approaches’. This will likely be beneficial for future-proofing the EU’s definition but could also lead to systems that are not commonly considered AI being regulated by the Act, potentially impacting innovation. As a result, the EU’s vision for a ‘Good AI Society’ has shifted from the narrow governance of robotics to an inclusive consideration of a variety of techniques and approaches. Additionally, the HLEG’s ethical principles and the AI Act now explicitly include accountability and transparency requirements, which represents a marked improvement for guiding the ethical development of AI.

Overall, the EU’s long-term vision for a ‘Good AI Society’, including the mechanisms for achieving it, appears coherent. The vision for governing AI is underpinned by fundamental European values, including human dignity, privacy and democracy. These individual rights, understood in a European sense, are not based on neoclassical, laissez-faire ideas of freedom, but rather seek to promote collective wellbeing and civil society.^11^ It has three practical cornerstones which are: improving economic outcomes, minimising social disruption, and developing appropriate ethical and legal frameworks. The risk-based approach, which combines hard and soft law, aims to ensure that harms to people are minimised, while allowing for the business and societal benefits of these technologies (Floridi et al., 2018). Importantly, guidance from the HLEG on how to develop AI systems ethically is non-binding and the AI Act is currently a proposed draft. The process of passing this draft into law requires confirmation from the European Parliament and Council, could take over 2 years and will likely be subject to negotiation, revisions and compromises. Accordingly, the vision for AI—which has largely been defined and outlined by the Commission—could still be altered by European MEPs, by Member State influence through the Council of Europe, and indirectly through private sector influence.

The EU’s domestic vision of a ‘Good AI Society’ involves inevitable trade-offs. The vision has been criticised for focusing too heavily on the protection of individual rights, as opposed to stimulating innovation, which could hinder economic growth and competitiveness (Brattberg et al., 2020). This criticism could be valid; however, if enacted properly, regulatory clarity and guidance could help foster ethical innovation in EU (Porter & van der Linde, 1995).

There are also gaps in the EU’s vision for a ‘Good AI Society’. Whilst references are made to the potential to use AI for socially good purposes like safeguarding the environment, the contribution of training AI models to increased greenhouse gas emissions is not addressed (Kaack, 2021; Cowls et al., 2021). This is particularly important considering the concentration of energy-intensive AI research and development occurring in North America and Europe, which contributes to the long-standing issue of environmental injustice (Ottinger, 2017). The outlined vision could also do more to support collective interests and social values; for example, through introducing a right to group privacy (Floridi, 2014; L. Taylor et al., 2017). Finally, the EU’s vision has given little consideration for how to address systemic risk, focusing on the risk to individuals from specific systems rather than the potential of AI to cause wider societal disruptions. As an example, the AI Act attempts to tackle individual manipulation from recommender systems, but does not consider that they could polarise online discourse more broadly (Whittlestone et al., 2021). It would be desirable for the EU (in line with its vision) to embrace the ethical use of AI for socially good purposes without exacerbating existing environmental problems or collective harms (Tsamados et al., 2021).

### International Policy

The EU’s international vision predominantly focuses on promoting cooperation in AI governance based on the respect for fundamental rights (European Commission, 2020a). However, it is also characterised by the extraterritorial scope of proposed measures. If passed, the AI Act would apply not only to providers established in the EU, but to anyone who places services with AI systems in the EU’s Single Market and where the output produced by an AI system is used in the EU. These measures could lead to a so-called ‘Brussels Effect’, which has already been seen with the enactment of the GDPR, whereby the market and regulatory power of the EU creates global standards that are followed by technology companies and in turn, adopted by third countries (Bradford, 2020).

The EU’s external-facing vision for a ‘Good AI Society’ should also be understood as part of the wider objective of ‘digital sovereignty’, for which AI is a constituent part (Floridi, 2020b; Roberts et al., 2021a). Though ‘digital sovereignty’ has not been clearly defined nor consistently used across EU institutions, it is generally understood in terms of maintaining strategic autonomy through having the capacity to determine its long-term social and economic future (Timmers, 2019). At present, this is threatened most significantly by American and Chinese technology companies, that are, as one European Parliament report put it, “increasingly seen as dominating entire sectors of the EU economy” (Madiega, 2020). Digital sovereignty is a policy agenda formulated in response to this challenge that aims at strengthening European control over external actors through improved regulatory means and the promotion of domestic innovation (Roberts et al., 2021a).

One notable omission in the EU’s outward-facing strategy is a developed position on the use of AI in the military domain. The proposed AI Act mentions AI for defence only to remark at the outset that the subject is not covered in the document, despite the fact that a number of Member States’ armies and security agencies have already begun to research and develop AI-enabled capabilities (Boulanin et al., 2020; Franke & Sartori, 2019). Several EU agencies such as the European Defence Agency (EDA), the Agency for Cybersecurity, and the Agency for the Operational Management of Large-Scale IT Systems in the Area of Freedom, Security and Justice, have opted so far for a cautious approach, sharing research, reports and recommendations, but no definite guidelines (ENISA, 2020; eu-LISA, 2020). Similarly, the European Parliament recently voted to adopt non-binding guidelines for military and non-military use of AI, focusing on the need to preserve human decision-making, respect human dignity and protect human rights (European Parliament, 2021). The much-anticipated “AI action plan” of the EDA that builds on requirements and preferences expressed by Member States is expected to provide clearer guidelines on the topic soon (EDA, 2020).

### Assessing Progress in Achieving the EU’s Good AI Society

The EU’s progress in achieving its high-level policy priorities is mixed. In regard to the first two overarching priorities outlined in the Communication, namely boosting industry capacity and preparing for social disruption, numerous initiatives have been introduced. There has been a concerted effort by both the Commission and Member States to invest heavily in the opportunities AI presents. For instance, the European Investment Bank (EIB) together with the European Investment Fund (EIF) pledged €150 million to support AI companies across Europe (European Commission, 2020b). Various policies have also been outlined that seek to mitigate the societal disruptions that AI can cause, including the Digital Education Action Plan (2021–2027) to ensure that citizens have robust digital skills in the face of market change, and introducing a Code of Practice on Disinformation for platforms to follow.

Significant progress has also been made towards the EU’s third priority of developing legal and ethical frameworks for the governance of AI. Specific guidance was developed for the HLEG’s principles and recommendations, which provides those developing and deploying AI with a checklist for deploying principles into practice. Although this guidance is voluntary, the proposed AI Act would provide enforceable regulation to support these ends.

Despite these efforts, the extent to which the EU’s vision of a ‘Good AI Society’ is being fully achieved is still questionable. The aim of boosting the EU’s industrial capacity is hamstrung by the current funding of the EU AI ecosystem, which has been criticised as being inadequate when compared to the US’s and China’s (Bughin et al., 2019). Moreover, some EU research funding—especially via Horizon 2020—has been criticised as being unethical because of the support provided to several projects using AI for law enforcement, despite potentially threatening fundamental EU values (Fuster & Brussel, 2020). This indicates that issues remain for the EU’s AI funding model.

A more fundamental question is whether the EU’s economic, social and regulatory aims will be achieved equally throughout Europe. Although the lack of EU funding is assuaged by that of certain European countries, including France, Germany and Sweden, this is not the case everywhere. This spending gap is symptomatic of a wider problem of ensuring an even spread of positive social and economic outcomes throughout the EU. Some Member States, typically in Western Europe, have developed AI strategies, yet this is mostly not the case in Eastern and Southern Europe (Brattberg et al., 2020). This is consequential for social outcomes, as different levels of investment lead to an unequal accrual of benefits as well as divergent levels of risk. For instance, Finland ranks 55 places higher than Croatia for AI readiness, measured by a number of metrics such as vision, infrastructure and data quality (Government AI Readiness Index, 2020, n.d.).

From a regulatory perspective, the proposed AI Act provides a strong foundation for standardising protections across Europe through prohibiting some use cases and providing common criteria for defining and regulating high and limited-risk AI. The AI Act proposes a European AI Board, made up of the heads of each Member States’ national supervisory authorities, which seeks to provide ongoing guidance for governing AI. Importantly, the proposed AI Act gives the Commission significant influence in regulating AI, such as through chairing the European AI Board.^12^ Some commentators have speculated that the proposal also gives the Commission power to intervene in national enforcement, if national authorities lack the expertise, resources or will to enforce the regulation’s measures (Reinhold & Müller, 2021). If this proposal comes to fruition, then it could ensure that the EU’s regulatory vision is enforced across Europe.

Even if enforcement is relatively standardised across the EU, there is a risk that the proposals may either ‘overregulate’ or ‘underregulate’ AI, depending on the standpoint taken.

In terms of banned systems, the proposed AI Act states that “certain AI systems intended to distort human behaviour, whereby physical or psychological harms are likely to occur, should be forbidden”. This clause is encompassing and could potentially include a range of use cases, such as recommender systems which are intended to nudge an individual to a specific kind of content whilst limiting their exposure to others (Milano et al., 2020). It could reasonably be argued that these systems cause mental and physical harms, such as through promoting ‘anti-vax’ content or encouraging extremism (as was arguably the case with the Capitol Riots) (Hao, 2021). Whilst recommender systems can undoubtedly prove harmful, prohibiting them would be a disproportionate response given the benefits they bring to everyday life when using digital services, such as buying products, listening to music, or watching films online. Moreover, without further clarification, overregulation could stymie innovation through increased costs and red tape, leading the EU to lose competitiveness against other states and in turn, hinder positive economic outcomes (Brattberg et al., 2020; Murgia & Espinoza, 2020).

At the same time, questions can be raised about the EU underregulating. Some members of the HLEG criticised the vague and non-committal nature of the Guidelines, which was blamed on regulatory capture by a substantial industry contingent that outweighed the number of ethicists on the board (Kelly, 2020). The AI Act may alleviate the concerns of non-committal enforcement, however, key weaknesses remain in measures. The ban on ‘real-time’ remote biometric surveillance has key exclusions, including for cases of missing children, terrorist threats or for serious crimes with prior judicial approval. In response to this, the European Data Protection Supervisor, Wojciech Wiewiórowski, stated that he was disappointed that the Act did not provide for a complete moratorium on remote biometric surveillance (European Data Protection Supervisor, 2021), as had been called for by a cross-party group of 40 MEPs a week earlier (Lomas, 2021).

Likewise, the requirements for providers of high-risk systems appears strict with an ex-ante conformity assessment needing to be completed which covers data, documentation, transparency, oversight, robustness and security. However, for most providers this is an *internal process* rather than a third-party document check. Companies are theoretically free to follow whichever standards they please, yet following harmonised standards developed by European bodies will likely be a cheaper and safer bet. On the surface, this might appear to be another check, however, relying on standards bodies to explicate the value-laden provisions of the draft AI Act is problematic, particularly given the high barriers to entry for interest groups to participate in the standards making process (Veale & Borgesius, 2021). Likewise, text surrounding disparate impact assessments are vague and non-committal, with little in the way of formal requirements for checks on bias (MacCarthy, & Propp, 2021). This is particularly problematic given the lack of specific redress mechanisms (Del Castillo, 2021). As a result, effective protection from high-risk systems will be largely reliant on interpretations by standards bodies and effective internal compliance by companies, which could lead to ineffective or unethical outcomes in practice. Indeed, precedent suggests that some of these potential limitations may materialise, particularly for smaller companies with fewer resources, with an EU-affiliated survey reporting that over half of small companies were not GDPR compliant in 2019 (Wolford, 2019). This raises a more general question over the effectiveness of a prescriptive regulatory approach for governing AI and achieving the EU’s vision (Clark & Hadfield, 2019).

A final uncertainty, likely to be influential in determining whether the EU is able to achieve its vision of a ‘Good AI Society’, is the extent to which the EU agenda of digital sovereignty is both articulated and successfully enacted. The loose aim of improving the EU’s ability to ‘act independently’ (Madiega, 2020; Von Der Leyen, 2020) fails to adequately address whom the EU is seeking to achieve independence from (i.e., which other actors might claim digital sovereignty) nor the extent to which choices *can* be made by the EU ‘independently’ (Roberts et al., 2021a). Without a clear articulation of the EU’s digital sovereignty agenda, it is difficult to pool support around a clear, holistic policy approach to the digital that can stimulate EU competitiveness beyond regulatory measures for AI.

## The US’s Approach to AI

In October 2016, the White House Office of Science and Technology Policy (OSTP) released the US’s first reports focusing specifically on AI: ‘Preparing for the Future of Artificial Intelligence’ and the ‘National Artificial Intelligence Research and Development Strategic Plan’. These reports defined the government’s role in the development of AI as a facilitator of innovation and a minimalist regulator and outlined how federal R&D investments would guide the “long term transformational impact of AI”. In their assessment of these policy documents, Cath et al. (2018) stressed that merely applying existing frameworks to new problems was inadequate.

The Trump administration continued the laissez-faire approach to AI laid out in these documents, and initially went further by stating that it had no intention of developing a national plan. This position was criticised on account of its failure to stimulate investment, nurture talent and minimise harms (Knight, 2018), and the administration backtracked to some extent with the signing of the American AI Initiative in February 2019. This executive order emphasised the importance of continued American leadership in AI and outlined five key principles:

1. Driving technological breakthroughs in AI to promote scientific discovery, economic competitiveness and national security.
2. Developing appropriate technical standards and reducing barriers to the safe testing and deployment of AI in order to enable the creation and adoption.
3. Training American workers with the skills to prepare them for jobs of the future.
4. Fostering public trust and confidence in AI technologies and protect civil liberties, privacy and American values.
5. Promoting an international environment that supports research and opens markets for American AI industries, while protecting the US’s technological advantage, including AI technologies from competitors.

Policy documents released since flesh out these five principles. In November 2020, as Trump’s own administration was coming to a close, the White House released guidance for government agencies proposing new AI regulations for the private sector, which centres around three themes: limiting regulatory overreach; ensuring public engagement; and promoting trustworthy AI that is fair, transparent and safe (Hao, 2020). These ideas are contained within a preamble, as well as 10 principles for the stewardship of AI applications (Table 2).

**Table 2:** US Guidance for Regulation of AI Principles (Executive Office of the President, 2020)

Public trust in AI	The government must promote reliable, robust and trustworthy AI applications
Public participation	The public should have a chance to participate in all stages of the rule-making process
Scientific integrity and information quality	Policy decisions should be based on science
Risk assessment and management	Agencies should decide which risks are unacceptable
Benefits and costs	Agencies should select approaches that maximise net benefits
Flexibility	Agencies should pursue a technology-neutral, flexible approach
Fairness and non-discrimination	Agencies should make sure AI systems do not discriminate illegally
Disclosure and transparency	Context-specific transparency measures are necessary for public trust
Safety and security	Agencies should promote AI systems that are safe, secure and operate as intended
Interagency coordination	Interagency cooperation and coordination is necessary for consistent policies

Most recently, the National AI Initiative Act of 2020 codified the US’s vision into law when it passed in early 2021. The aims of the Act largely reflect those of the American AI Initiative, centring on American AI leadership in R&D and the development of trustworthy AI systems, as well as preparing for potential workforce disruptions and coordinating military and civilian sectors. It also mandates the establishment of bodies to provide federal-level guidance, including a ‘National Artificial Intelligence Office’, which is tasked with supporting AI R&D, educational initiatives, interagency planning and international cooperation; an expert National AI Advisory Committee for assessing the degree to which the US is fulfilling its aims; and a subcommittee for AI and law enforcement that advises on issues such as legality, biases and data security.

### Domestic Policy

The US’s vision for a ‘Good AI Society’ is characterised by an acute focus on limiting regulatory overreach. However, the National AI Initiative Act signals a growing recognition of the need to provide some coordination and support for the US to fulfil its ambitions. Nonetheless, rather than foregrounding regulations that protect individual freedoms, in the sense of negative liberty, the US strategy centres on empowering positive liberty of individuals and corporations to benefit from AI.

A largely laissez-faire approach to the governance of AI technologies may be permissible from an economic perspective, though the assumption that limiting regulation is the best way of ensuring innovation has been questioned by experts, including those who previously advised the Obama administration on AI (Johnson, 2020). Ethically, however, the US’s vision is problematic given the numerous ethical challenges linked to the pervasive distribution of AI technologies. The White House’s emphasis on avoiding overregulation is a clear disincentive for federal agencies to introduce rigorous regulations, and White House officials have previously criticised states that have considered banning facial recognition technology (Vincent, 2020a). Whilst self-regulation by industry may mitigate some potential ethical harms, the lack of specific regulatory measures and oversight can lead to practices such as ethics washing (introducing superficial measures), ethics shopping (choosing ethical frameworks that justify actions a posteriori) and ethics lobbying (exploiting digital ethics to delay regulatory measures) (Floridi, 2019). In practice, the inadequacy of private sector regulatory measures has facilitated numerous harms, such as from biases in facial and emotion recognition technologies leading to discrimination (Buolamwini & Gebru, 2018; Crawford, 2021). To see the potential for wider harms that this approach may cause, one only needs to look at previous failures of self-regulation without adequate oversight in other high-risk areas, such as the self-regulation of credit agencies leading up to the 2007–2008 financial crisis and of the airline industry before the Boeing 737 crashes (Clark & Hadfield, 2019).

### International Policy

In contrast to this *hands-off* approach on the domestic stage, the US’s vision appears geared towards a *hands-on* approach to the unilateral^13^ governance of AI internationally. The American AI Initiative states the need to promote an international environment that opens markets for American AI industries, protects the US’s technological advantage and ensures that international cooperation is consistent with ‘American values’. In doing so, this vision incorporates mercantile undertones.

Within this overarching vision, the US’s AI strategy for defence is perhaps the most developed aspect. The 2019 ‘National Defense Authorization Act’ established the National Security Commission on AI (NSCAI), an independent commission set up to review advances in AI to address the national security needs of the US. In its final report, the NSCAI concluded that “[t]he United should invest what it takes to maintain its innovation leadership, to responsibly use AI to defend free people and free societies, and to advance the frontiers of science for the benefit of all humanity” (Schmidt et al., 2021, p. 14), and made a number of recommendations for achieving these ends.

Ethical principles for the use of AI in defence, which seek to ensure the compliance of AI applications with International Humanitarian Law while reinforcing national security, were issued by the US Defense Innovation Board in February 2020. The identified principles—according to which uses of AI in defence should be responsible, equitable, traceable, reliable and governable—have been defined considering AI as a defence capability that is essential for maintaining technological, and therefore operational, superiority over the adversary. The development of these ethical principles is a positive step forward; practically, however, their framing can be called into question. A push to ensure superiority over adversaries can lead to the development of advanced autonomous capabilities that lack meaningful control, something that the US risks with the introduction of systems such as the ‘Golden Horde’ munitions programme (Insinna, 2021). Accordingly, the framing of these ethical principles themselves creates tension with the stated desire for ethical outcomes.

Considering the US’s outward-facing AI strategy raises a number of questions over its efficacy. Although the US’s strategy of export bans may slow China down, programmes such as ‘Made in China 2025’ and ‘China Standards 2035’ seek to improve the domestic creation of emerging technologies and international technology standards respectively. As a result, export controls to China are no guarantee of prolonged US AI leadership. For instance, Huawei is making rapid progress on a chip plant that will eschew American technology, allowing them to skirt US sanctions and maintain their chip supply (Hille et al., 2020). These policies could just speed up China’s effort to develop and export competing capacities, though evidence of China’s success in developing domestic chip capabilities has thus far been mixed (Khan et al., 2021). Similarly, the absence of a developed domestic approach for promoting responsible innovation could harm the US’s international competitiveness (Rasser et al., 2019). For example, whilst China is luring back expats through incentive schemes such as the Thousand Talents Programme, which could improve its overall competitiveness (Joske, 2020), the US has enacted hostile visa policies which could prove detrimental for AI firms (Coles, 2020).

Finally, it should be stressed that ‘American values’ are ill-defined. The website for Trump’s AI initiative seems to define ‘American values’ as “freedom, guarantees of human rights, the rule of law, stability in our institutions, rights to privacy, respect for intellectual property, and opportunities to all to pursue their dreams” (*Artificial Intelligence for the American People*, n.d.), the executive order considers privacy as separate from ‘American Values’, and other policy documents also define them differently. External protectionism contrasts with internal classical liberalism, with these inconsistencies raising questions about how unified the American approach to the governance of AI is within the federal government, especially compared to the more coherent value-set that underpins the EU’s ‘Good AI Society’ vision. As we will discuss in Sect. “Transatlantic Cooperation on AI Governance”, the Biden administration may be trying to create a more consistent definition of ‘American values’.

### Assessing Progress in Achieving the US’s Good AI Society

Several steps have been taken to materialise the US’s vision of a ‘Good AI Society’. In terms of promoting economic competitiveness and achieving R&D breakthroughs, the government has committed to double federal non-defence R&D AI funding in the 2021 fiscal year (Office of Science & Technology Policy, 2020). In August 2020, the US announced $1 billion in funding for 12 research hubs that focus on AI and quantum computing (Vincent, 2020b). Importantly, government funding only forms part of a dynamic AI ecosystem in which private sector innovation plays a central role. Though exact figures are difficult to ascertain, many estimates indicate that the US is the leading country in terms of private sector investment (*The Global AI Index*, n.d.). Despite these developments, some commentators argue that the government is not doing enough to stimulate the AI ecosystem (Rasser et al., 2019).

Efforts to prepare the US for societal change through educational and training initiatives are also growing. For instance, policy documents have been released which seek to improve lifetime STEM education (National Science and Technology Council, 2018) and enhance R&D through a number of research fellowships and training programmes (*Artificial Intelligence for the American People*, n.d.). However, the extent to which such programmes can resolve the longer-term societal changes that AI might cause should be questioned, particularly in regard to the less-educated parts of the population. In 2018, the US was ranked 9th out of 25 advanced economies in terms of readiness for automation, with vocational technical training considered inadequate (Paquette, 2018). Other studies echo this finding, pointing to the US as having weaker problem-solving skills in technology-rich environments than a number of other developed countries, with suggestions made that further lifelong training programmes are needed (Cummins et al., 2019).

When it comes to developing the types of AI that may lead to radical change in the job market, the lack of focus on ensuring equitable development may compound these issues. The National Science and Technology Council’s ‘Charting a Course for Success: America’s Strategy for STEM Education’ has “Increase Diversity, Equity, and Inclusion in STEM” as one of its pillars, but neither this document nor other AI-focused documents emphasise maintaining diversity throughout AI development, raising questions about its effectiveness at retaining underrepresented groups through the AI pipeline. Failing to focus on equity in training and retention across racial and socioeconomic groups could cause the societal changes linked to AI to be detrimental for already marginalised groups that may not be served by education and retraining initiatives. The National Academies’ artificial intelligence impact study on the workforce, mandated as part of the National AI Initiative Act, could shed more light on current workforce failings and future needs, though it is imperative that any study considers disparate impacts.

Federal efforts to ensure that AI is safe and trustworthy have been limited, with the aforementioned high-level principles the main existing action. These principles are merely guidance and the emphasis on ethical AI is outweighed by the clear prioritisation of a laissez faire regulatory approach. More recently, the Government Audit Office released an AI Accountability Framework for use by federal agencies and other entities (*Accountability Framework*, 2021), which represents a promising, albeit voluntary, development. Another notable development is a 2021 blog post by the Federal Trade Commission, which stated that it prohibited unfair practices and that this *included* racially biased algorithms (Jillson, 2021). This is suggestive of the potential for federal enforcement against biased systems. Nonetheless, these examples of new measures and reinterpretations of existing law are the exception rather than the norm amongst federal agencies.

In contrast to a hands-off approach by the federal government, a handful of US cities have introduced their own measures to mitigate potential ethical harms of AI. Recent high-profile examples include San Francisco’s 2019 moratorium on government use of facial recognition technology and Portland’s private sector ban of the same technologies (Simonite, 2020). In the absence of clear national regulations, these are positive developments for achieving ethical outcomes within these states, however, local regulatory measures do not prohibit federal use and are leading to significant gaps in standards between states.

The US has made considerable progress in promoting an international environment that is beneficial for its vision of a ‘Good AI Society’. It has ensured open markets by pushing back against data localisation measures that restrict the flow of data, by asserting pressure both formally in trade deals and also informally through public threats and increased diplomatic coercion (Basu, 2020; Sherman, 2020). Steps have also been taken to counter strategic competitors, including export restrictions on numerous AI products in an effort to keep certain technologies out of China’s hands for supposed economic and security reasons (‘U.S. Government Limits Exports of Artificial Intelligence Software’, 2020). Combined, these measures have allowed the US to maintain open markets for its own products, whilst hampering the efforts of competitors to overtake.

Despite this, some scholars continue to argue that the US has not gone far enough in protecting its AI capacities, including its data sets and stopping the illicit transfer of technologies (Rasser et al., 2019; Thomas, 2020). The criticism is that the US has not adequately protected its technological and economic leadership, nor prevented ethically concerning uses of its technologies for repression elsewhere, such as the surveillance-enabled persecution of the Uyghur minority in Xinjiang, China (Mozur & Clark, 2020). In fact, the US is one of the most prominent exporters of surveillance technology globally.^14^

## Transatlantic Cooperation on AI Governance

Both visions for a ‘Good AI Society’ emphasise the importance of international dialogue, and each government has already taken steps to improve international governance efforts. After the US initially refused to join the Global Partnership on AI (GPAI)—an international and multi-stakeholder initiative to guide the responsible development and use of AI—due to a fear that the ethics focus would hinder innovation, it joined, like the EU and others, in May 2020. This was predominantly an attempt to counter the perceived threat of China and ensure that AI develops in line with ‘American values’ globally. Shortly after, in September 2020, the US took an active step at promoting defence cooperation through establishing the AI Partnership for Defense. This initiative seeks to share lessons, improve interoperability and increase data sharing amongst allies. Importantly, the initiative includes many European countries, such as Denmark and France, but excludes others, including Germany and the Netherlands (Trabucco, 2020).

The EU and US also support the OECD’s AI ethics principles, which centre on developing trustworthy AI based on five key values: sustainable growth and wellbeing, human-centred and fair, transparent and explainable, robust and safe, and accountable (OECD.AI, n.d.). Other international organisations are also trying to promote international and agreement on AI, such as UNESCO, whose draft Recommendation on the Ethics of AI has been provisionally accepted by member states (*AI Ethics*, 2021). However, given the 2018 withdrawal from UNESCO by the US, it seems unlikely that they will accept these measures.

Since Joe Biden’s inauguration in January 2021, there have been signs of the potential for deeper transatlantic ties, with the new president promising an American return to multilateralism and cooperation to defend democratic values (Schaake & Barker, 2020). In fact, technology is central on the agenda of the ‘summit of democracies’ that Biden has promised to convene, and there is a call to use such opportunity for establishing a dialogue on good governance that is grounded in shared values such as human rights and democracy. Similar calls have been made by EU for greater cooperation on technology governance (Hanke Vela & Herszenhorn, 2020). The website for Biden’s AI initiatives defines ‘American values’ explicitly, indicating the possibility of a more coherent foundation for future governance efforts like that of the EU, and emphasises that the US will work with allies to foster its goals (International Cooperation, 2021).

Firmer statements by figures in the Biden administration on high-risk technologies could create fertile ground for greater cooperation with Europe. For instance, as a candidate for president, Vice President Kamala Harris showed concern regarding the potential problems of using AI in the criminal justice system, and explicitly committed to “ensure that technology used by federal law enforcement […] does not further racial disparities or other biases” (Harris, 2019). With Harris now in office, this appears to be a promising foundation for deeper agreement in defining international AI ethics beyond the OECD’s high-level principles.

In reality, expectations on deep current or future transatlantic cooperation should be tempered. Assessing the international-facing strategies of the EU and the US reveals tensions that may be detrimental for international cooperation. Both the EU and US can be seen to be exporting their ideological approaches to AI beyond their domestic jurisdiction (albeit indirectly in the EU’s case). Indeed, the EU’s desire for digital sovereignty, which largely centres on curtailing American technology companies, is in direct conflict with the US agenda for ‘digital free trade’ and low barriers to entry for its corporations. It seems unlikely that these underlying positions will shift, given fundamental differences in areas such as digital trade which predate the Trump administration (Azmeh & Foster, 2016; Jones et al., 2021). Even within the short time that Biden has been president, there have been discussions of retaliatory tax measures against states which seek to levy taxes on American technology companies (Islam, 2021).

Likewise, support from both the EU and US for the OECD’s ethical principles should not be read as alignment on what trustworthy AI should look like. Indeed, there has been a general coalescing around five ethical principles for governing AI within the West (Floridi & Cowls, 2019). Yet without a clear definition of the underpinning values system on the one hand and clear recommendations on the other, AI principles alone provide little indication how ethical AI will be developed and used and of what a Good AI Society looks like (Roberts et al., 2021b).

As the ethical approaches of the EU and the US further develop, it is likely that differences in underlying values will become clearer, both in terms of how principles are understood and enforced. This has already been the case with the interpretation of ‘human-centred values’, including privacy. The EU has far more stringent data protection laws than the US, with the distinction between the two typified by the European Court of Justice’s judgment in the July 2020 case of *Data Protection Commissioner v. Facebook Ireland Ltd and Maximillian Schrems*, which ruled that the data protection provided by the EU-US Privacy Shield, a framework for regulating the exchanges of data between the EU and the US, was inadequate (Tracol, 2020).

## Conclusion

The EU and US have made significant progress since 2018 in solidifying their visions for what a ‘Good AI Society’ should look like and setting out paths forward for achieving these visions. These different visions—prioritising individual citizens in the EU versus national competitiveness of the US—have important consequences for the ethical governance of AI both domestically and internationally. From the standpoint of domestic AI governance, the EU’s approach is ethically superior. It foregrounds protecting citizens’ rights through outlining the guiding value of human-centric trustworthy AI, which anchors additional ethical principles. The enactment of the proposed AI Act would solidify this lead in ethical governance through providing a robust enforcement mechanism for high-risk uses of AI. It is evident that many of the fundamental values that should be at the heart of a ‘Good AI Society’, such as protecting human rights and preserving privacy, are present and will likely be protected through the enforcement of existing measures (e.g. the General Data Protection Act) and the introduction of new ones (e.g. the draft AI Act). This enforcement will undoubtedly be imperfect, but the measures in place will effectively guide good behaviours and penalise the worst offenders.

This is not to say that the EU’s vision cannot be improved upon; the inclusion of group privacy protections and more explicit measures to curtail the potential environmental harms of AI would help in achieving ethical outcomes through the improved protection and promotion of fundamental values. More generally, the EU’s regulatory framework also needs to be accompanied by stronger measures that promote responsible innovation to guarantee ethical outcomes. The EU’s relative lack of investment and leading technology companies has a knock-on effect into other areas, such as the attraction of AI talent.^15^ These deficiencies undermine the ability of the EU to ensure that technologies are developed and deployed in line with European values, as evidenced by the difficulties EU countries faced in developing and using domestic contact tracing applications in the COVID-19 pandemic (Sharon, 2020). Accordingly, the promotion of European competitors in the field of AI, including underlying and complementary technologies such as microelectronics, cloud, supercomputing and cybersecurity are needed to ensure that the EU’s vision of a ‘Good AI Society’ can materialise in practice.

The laissez-faire path taken by the US at a domestic level is more ethically questionable. It has largely placed the governance of AI in the hands of the private sector, leaving significant scope for organisations to prioritise their own interests over that of citizens. Whilst some of the measures outlined in the National AI Initiative Act appear to be a promising step in the right direction, an emphasis on ethical and regulatory measures is largely secondary to further strengthening R&D to improve competitiveness. Without government regulatory oversight, there is a significant risk of harm through ethics washing or shopping, with the effectiveness of private sector principles questionable (Hagendorff, 2020). Market mechanisms, such as pressure from consumers, employees and investors may check some unethical practices (Cihon et al., 2021). Indeed, some commentators have been positive about the establishment of AI ethics boards within big American technology firms (Newman, 2020). However, the firing of Timnit Gebru and Margaret Mitchell from Google (Vincent, 2021) reveals the limits to soft checks when the ethical permissibility of fundamental company practices are challenged. As such, some ‘American values’ may be protected by a laissez-faire vision of AI, in the sense that free market principles are preserved, yet this may come at the cost of protecting other fundamental values, such as human rights (including those enshrined in the US constitution). Given the significant risks that AI poses to fundamental values if unchecked, ranging from systemic discrimination to fostering monopolistic practices to exacerbating climate change, this approach is inadequate.

One continued risk for both the EU and US, if new measures are not introduced, is internal fragmentation. The benefits and risks associated with AI and data-driven technologies are already being spread unevenly across both the EU and the US, on account of different regulatory protections and opportunities from AI that are afforded to citizens. Such a fractious implementation is problematic as it will lead to uneven enforcement and unequal protection of the rights of citizens. This undermines the ability to maintain and project a consistent vision of a ‘Good AI Society’ from a governance perspective and raises ethical questions over whom is left unprotected by fractious regulations.

Whilst not irreconcilable, and despite the change of administration in the US, it is unlikely that these two visions will coalesce around deep transatlantic cooperation. In fact, as the EU’s digital sovereignty agenda continues to develop, increased friction with the US and its technology companies may be anticipated. This is not to say that deeper transatlantic cooperation is impossible. For instance, if relations between China and both the US and the EU continue to deteriorate, then it is possible that more substantive engagement in areas such as defence will materialise between them. Nonetheless, it would be desirable for closer alignment to instead emerge through the US turning its statements about ethical AI into reality. For example, the statement by the Federal Trade Commission that it will consider enforcing against unfair or deceptive AI could provide a strong starting point for other agencies to announce their own measures, but this alone is inadequate. Enforceable AI regulations, meaningful anti-trust measures and a cooperative international environment, both with allies and competitors, are all necessary for a truly ‘Good AI Society’ to materialise for all those who are part of it. How these developments play out in the next few years will have a significant influence on the development and deployment of AI and the protections that are afforded to citizens in the United States, Europe and elsewhere .

## Footnotes

1 This is not true of all comparative analyses. For instance, Feijóo et al. (2020) outline the AI policies of ‘major players’ and consider how an alignment of policy can be facilitated, whilst Ulnicane et al. (2021) analyse 49 AI policy documents to consider how governance has been framed.

2 There are many types of ethical pluralism which vary in terms of how much similarity there is between different values systems, see (Ess, 2020b). In our view, there is a universal moral minimum which provides individuals with chance to engage and connect; see (Ess, 2006, 2020b).

3 Since 1988, the United Nations (UN) General Assembly has adopted at least one resolution annually dealing with some aspect of democracy (*Democracy*, n.d.).

4 See Walzer (1994).

5 See C. Ess (2005); Ma (2019); Reviglio & Alunge (2020).

6 See the UN’s Universal Declaration on Human Rights and texts by scholars such as Chan (1999) and C. Taylor (1996).

7 The Paris Agreement, for instance, is a legally binding international treaty on climate change that has been adopted by 196 parties (*U.N. Doc. FCCC/CP/2015/L.9/Rev/1*, 2015).

8 Disclosure: LF participated in the meeting.

9 The remaining Member States, Greece, Cyprus, Romania and Croatia, signed the declaration later in the year.

10 Disclosure: LF was a member.

11 https://www.pewresearch.org/global/2011/11/17/the-american-western-european-values-gap/.

12 This is a change from the European Data Protection Board (EDPB) that was mandated in the GDPR to fulfil a similar function, with the chair of the EDPB elected from national supervisory authorities.

13 As we will discuss in the next section, the US was initially hesitant to join multistakeholder efforts at governing AI but has since shifted to engaging more collaboratively.

14 https://carnegieendowment.org/publications/interactive/aI-surveillance.

15 https://macropolo.org/digital-projects/the-global-ai-talent-tracker/.
